# Pivotal trials of orthopedic surgical devices in the United States: predominance of two-arm non-inferiority designs

**DOI:** 10.1186/s13063-017-2032-2

**Published:** 2017-07-24

**Authors:** S. Raymond Golish

**Affiliations:** Department of Surgery, Jupiter Medical Center, 1210 S. Old Dixie Hwy, Jupiter, Palm Beach, FL 33458 USA

**Keywords:** Orthopedic surgery, Medical devices, Orthopedic surgical devices, Spinal devices, United States Food and Drug Administration, Randomized clinical trials, Pivotal trials, Non-inferiority trials, Two-arm trials

## Abstract

**Background:**

The United States Food and Drug Administration (FDA) reviews class III orthopedic devices submitted for premarket approval with pivotal clinical trials. The purpose of this study was to determine the types of orthopedic devices reviewed, the design of their pivotal clinical trials, and the subjective factors affecting the interpretation of clinical trial data.

**Methods:**

Meetings of the FDA Orthopaedic and Rehabilitation Devices Panel were identified from 2000–2016. Meeting materials were collected from FDA electronic archives and notes were made regarding the device-type and subsequent approval and recall, the design of pivotal clinical trials, and issues of trial interpretation debated during panel deliberations.

**Results:**

The panel was convened on 29 separate occasions over the course of 35 days to deliberate 38 distinct topics. Of these, 23 topics included clinical data submitted for approval of a device, and two topics were excluded. Of the 23 devices, five were biologic, three were hip arthroplasty, three were disc arthroplasty, two were viscosupplementation, three were interspinous process devices, and seven were other devices. Of the 23 pivotal trials, 20 (87.0%) were randomized controlled trials (RCTs), consisting of 13 (65.0%) non-inferiority trials and 7 (35.0%) superiority trials, and all RCTs were two-arm trials. At panel, the most commonly debated issues were related to the design and interpretation of non-inferiority trials.

**Conclusions:**

A broad array of device types is reviewed by the FDA. The predominance of two-arm non-inferiority trials as pivotal studies indicates that the nuances of their design and interpretation are commercially important.

## Background

Orthopedic surgery is one of the largest segments of the medical device industry. In the United States jurisdiction, orthopedic devices are regulated by the Division of Orthopedic Devices, Office of Device Evaluation, Center for Devices and Radiologic Health of the Food and Drug Administration (FDA). With few exceptions, many of the most complex, novel, or high-risk orthopedic devices (class III devices) are subject to premarket approval (PMA), the FDA’s most intensive review process for devices [1]. Typically, a PMA application will include results from one or more clinical trials conducted under investigational device exemption (IDE) status.

The FDA utilizes approximately 50 committees and panels that provide expert advice on matters of science and policy. Of these, the Orthopaedic and Rehabilitation Devices Panel provides advice on orthopedic devices, and PMA applications may be subject to review in a public forum. Though not all PMAs come before the panel (and not all panel meetings review PMAs), the PMA process including panel review represents the most intensive scrutiny and highest level of scientific deliberation that an orthopedic device may receive in consideration of regulatory approval.

The purpose of the present study was to assess the design of pivotal trials of orthopedic devices and their interpretation at FDA panel meetings. This is important because IDE trials are frequently the best scientific evidence available for both regulatory affairs and clinical decision making. The relationship of device characteristics to the rate of device approval and recall was studied, since these are mechanismfigurs by which devices enter and exit the market for routine clinical use. Similarly, the characteristics of the IDE trials were reviewed including the trial designs and their relationship to approval and recall. Finally, those clinically relevant aspects of trial structure that affect panel deliberations were identified.

## Methods

Meetings of the Orthopaedic and Rehabilitation Devices Panel were identified from the beginning of the 2000 calendar year to 2016 from official announcements in the Federal Register. Meeting materials were obtained from the FDA electronic archives, including panel-packs, sponsor executive summaries, FDA executive summaries, rosters, postmeeting summaries, and official meeting transcripts. Notes were made regarding the purpose of the meeting as either reclassification, special advisory, PMA, or premarket notification (i.e., 510(k)).

For PMA and 510(k) topics, IDE trials were classified as a randomized clinical trial (RCT) versus observational/nonrandomized study, and superiority versus non-inferiority design. The number of arms and the type of control were recorded. Notes were made regarding comments and questions of panelists regarding complexities in the interpretation of clinical trial data, as reflected in the official transcripts when available. Comments were qualitatively categorized into three major categories: outcome measures, randomized trial conduct, and issues specific to non-inferiority trials. Among outcome measures, subtopics included time points, primary composite endpoint, secondary endpoints, surrogate radiographic endpoints, choice of clinical instruments, and time of final follow-up. For randomized trials, subtopics included power, randomization ratio, blinding, number of arms, choice of control procedure, and rate of crossover, dropout, and missing data. For issues specific to non-inferiority trials, subtopics included the choice of active control, the choice of non-inferiority margin, assay sensitivity, and analysis of datasubsets (i.e., intention-to-treat (ITT), modified ITT, as-treated (AT), and per-protocol (PP) analyses).

For each device, a note was made of the device type—biologic, hip arthroplasty, disc arthroplasty, viscosupplementation, interspinous process device, or other device (all devices with only one example per type). The subsequent approval and recall of devices was noted up to the time of writing. The date of each conducted panel meeting was noted. For the purposes of hypothesis testing, the number of meetings per year was analyzed by dividing the investigation period (2000–2016) into two roughly comparable portions—years 2000–2008 and 2008–2016 inclusive. The rate of device approval was tested with the rate-ratio test for the intensity of Poisson processes. The subsequent approval and recall of devices was investigated up to the time of writing. For the purposes of hypothesis testing, devices were grouped in four dichotomous ways: spinal versus nonspinal, arthroplasty versus nonarthroplasty, biologic versus nonbiologic, and other devices versus non-other. Hypotheses were tested with the Fisher exact test.

## Results

Between 20 July 2000 and 20 April 2016, the panel was convened on 29 separate occasions over the course of 35 days to deliberate 38 distinct topics. Table 1 summarizes the panel meetings. Of these 38 topics, 25 involved the review of clinical data submitted by a sponsor seeking regulatory approval of a particular device. Two topics were excluded, yielding 23 unique devices with IDE trial data. One excluded topic was a re-review of a previously reviewed device, which was excluded because the device was previously approved and subsequently unapproved under controversial circumstances [2, 3] The second excluded topic was an atypical PMA application for two related but distinct ceramic hip arthroplasty devices that had undergone a joint clinical trial. The panel split the devices into two topics, voting one up and one down [4], though the entire PMA was subsequently approved [5].

**Table 1:** Topics of the Orthopaedic and Rehabilitation Devices Panel 2000–2016

Number	Date	Sponsor	Device
1	April 20, 2016	Cartiva	Synthetic Cartilage Implant [20]
2	February 19, 2015	Medtronic	DIAM Spinal Stabilization System [38]
3	February 20, 2015	Vertiflex	Superion Spinous Process Spacer [16]
4	February 21, 2014	N/A	Iontophoresis [39]
5	December 12, 2013	N/A	Spinal Spheres [40]
6	December 12, 2013	N/A	Stair climbing wheelchairs [41]
7	December 12, 2013	N/A	Mechanical wheelchairs [42]
8	May 22, 2013	N/A	Pedicle screw spinal systems [43]
9	May 21, 2013	N/A	Nonthermal short wave diathermy [44]
10	September 21, 2012	N/A	Posterior cervical pedicle/lateral mass screw spinal systems [45]
11	June 27–28, 2012	N/A	Metal-on-metal hip implant systems [31]
12	May 12, 2011	BioMimetic	Augment Bone Graft [6]
13	May 27, 2010	Medtronic	Amplify rhBMP-2 Matrix [7]
14	March 23, 2010	ReGen Biologics	Collagen Scaffold (CS)/Menaflex
15	November 4, 2009	Zimmer Spine	Dynesys Spinal System [8]
16	August 19, 2009	Q-Med	Durolane [46]
17	August 18, 2009	DePuy Orthopaedics	CoMplete Acetabular Hip System [47]
18	March 31, 2009	Stryker Biotech	OP-1 Putty [48]
19	December 9, 2008	Genzyme	Synvisc One [49]
20	November 14, 2008	ReGen Biologics	Collagen Scaffold (CS)/Menaflex
21	July 15, 2008	FzioMed	Oxiplex/SP Gel [50]
22	July 17, 2007	Medtronic	Bryan Cervical Disc [51]
23	April 24, 2007	Link America	Scandinavian Total Ankle Replacement (STAR) System [52]
24	February 22, 2007	Corin USA	Cormet 2000 Hip Resurfacing System [53]
25	September 19, 2006	Medtronic	Prestige Cervical Disc System [29]
26	June 2, 2006	RS Medical	Non-invasive Bone Growth Stimulator [54]
27	September 8, 2005	Smith & Nephew	Birmingham Hip Resurfacing System
28	September 9, 2005	N/A	Design of clinical studies for spinal devices
29	August 31, 2004	St. Francis Medical	X STOP Interspinous Process Decompression System [30]
30	June 3, 2004	OSMA	Knee mobile bearing and unicompartmental arthroplasty [55]
31	June 3, 2004	OSMA	Total knee and total hip [55]
32	June 2, 2004	DePuy Spine	Charité Artificial Disc [28]
33	December 11, 2003	N/A	Intervertebral body fusion device [56]
34	November 21, 2002	Wyeth	InductOs rhBMP-2/absorbable collagen sponge [57]
35	November 20, 2002	Independence	iBOT 3000 Mobility System [58]
36	January 10, 2002	Medtronic	InFUSE rhBMP-2/absorbable collagene sponge [59]
37	July 20, 2000	HealthTronic	OssaTron Lithotripter [4]
38	July 20, 2000	Howmedica	ABC and Trident Acetabular Systems [4]

Of the 23 unique devices with pivotal IDE trial data, 20 (87.0%) were RCTs, consisting of 13 (65.0%) non-inferiority trials and seven (35.0%) superiority trials. The remaining three (13.0%) studies were observational/non-randomized. All non-inferiority studies (13) were two-arm trials versus an active control, and none was a three-arm study including a sham control. Table 2 summarizes the PMA topics and clinical trial structures.

**Table 2:** Premarket approvals with investigational device exemption trials 2000–2016

Number	Date	Sponsor	Device		
1	April 20, 2016	Cartiva	Synthetic Cartilage Implant		
2	February 19, 2015	Medtronic	DIAM Spinal Stabilization System		
3	February 20, 2015	Vertiflex	Superion Spinous Process Spacer [16]		
4	May 12, 2011	BioMimetic	Augment Bone Graft [6]		
5	May 27, 2010	Medtronic	Amplify rhBMP-2 Matrix [7]		
6	November 4, 2009	Zimmer Spine	Dynesys Spinal System [8]		
7	August 19, 2009	Q-Med	Durolane [46]		
8	August 18, 2009	DePuy Orthopaedics	CoMplete Acetabular Hip System [47]		
9	March 31, 2009	Stryker Biotech	OP-1 Putty [48]		
10	December 9, 2008	Genzyme	Synvisc One [49]		
11	November 14, 2008	ReGen Biologics	Collagen Scaffold (CS)/Menaflex		
12	July 15, 2008	FzioMed	Oxiplex/SP Gel [50]		
13	July 17, 2007	Medtronic	Bryan Cervical Disc [51]		
14	April 24, 2007	Link America	Scandinavian Total Ankle Replacement (STAR) System [52]		
15	February 22, 2007	Corin USA	Cormet 2000 Hip Resurfacing System [53]		
16	September 19, 2006	Medtronic	Prestige Cervical Disc System [29]		
17	September 8, 2005	Smith & Nephew	Birmingham Hip Resurfacing System		
18	August 31, 2004	St. Francis Medical	X STOP Interspinous Process Decompression System [30]		
19	June 2, 2004	DePuy Spine	Charité Artificial Disc [28]		
20	November 21, 2002	Wyeth	InductOs rhBMP-2/absorbable collagen sponge [57]		
21	November 20, 2002	Independence	iBOT 3000 Mobility System [58]		
22	January 10, 2002	Medtronic	InFUSE rhBMP-2/absorbable collagene sponge [59]		
23	July 20, 2000	HealthTronic	OssaTron Lithotripter [4]		
Number	Filing	Design	Type	Arms	Control
1	PMA (P150017)	RCT	Non-inferiority	2	Active
2	PMA (1400007)	RCT	Superiority	2	Usual care
3	PMA (P140004)	RCT	Non-inferiority	2	Active
4	PMA (P100006)	RCT	Non-inferiority	2	Active
5	PMA (P050036)	RCT	Non-inferiority	2	Active
6	PMA (P070031)	RCT	Non-inferiority	2	Active
7	PMA (P060013)	RCT	Superiority	2	Sham
8	PMA (P090002)	RCT	Non-inferiority	2	Active
9	PMA (P060021)	RCT	Non-inferiority	2	Active
10	PMA (P940015)	RCT	Superiority	2	Sham
11	510(k) (K082079)	RCT	Superiority	2	Active
12	PMA (P070023)	RCT	Superiority	2	Active
13	PMA (P060023)	RCT	Non-inferiority	2	Active
14	PMA (P050050)	RCT	Non-inferiority	2	Active
15	PMA (P050016)	Non-randomized	Non-inferiority	2	Active
16	PMA (P060018)	RCT	Non-inferiority	2	Active
17	PMA (P040033)	Observational	–	–	–
18	PMA (P040001)	RCT	Superiority	2	Usual care
19	PMA (P040006)	RCT	Non-inferiority	2	Active
20	PMA (P000054)	RCT	Superiority	2	Active
21	PMA (P020033)	Observational	Crossover	2	Usual care
22	PMA (P000058)	RCT	Non-inferiority	2	Active
23	PMA (P990086)	RCT	Superiority	2	Sham

*IDE* investigational device exemption, *PMA* premarket approval, *RCT* randomized controlled trial

**Fig. 1:**
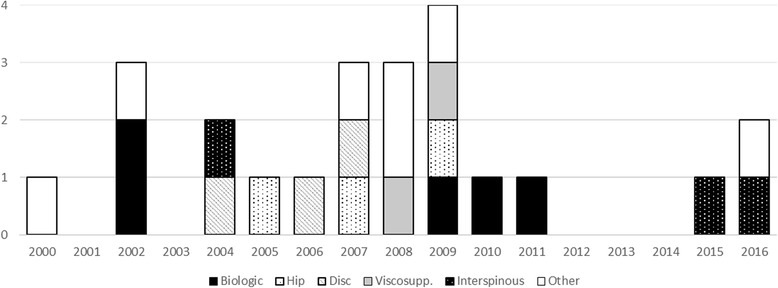
IDE trials by device class by year

In total, five devices were biologic, three were hip arthroplasty, three were disc arthroplasty, two were viscosupplementation, three were interspinous process devices, and seven were other devices. Figure 1 presents the number of panel meetings by device type per year. The rate of meetings per year for the matched periods from 2000–2008 was compared to years 2008–20016. For the earlier period, the rate was 1.47 meetings per year versus 1.24 meetings per year for the later period. This decrease of 16% over the study period is not statistically significant (*p* > 0.05). The results do not change by excluding the middle year 2008 or by assigning it to the earlier or later period.

Of 23 devices, the approval status was known for 21 as of the time of writing, as approval decisions and their disclosure takes time from the date of the panel meeting. Of these 21 devices, fourteen devices were approved subsequent to panel review (66.7%), and no device directly reviewed by the panel was subsequently recalled. The clinical trial type of superiority versus non-inferiority was not significantly associated with approval (*p* > 0.05). Regarding device type, there was no significant difference in the rate of approval between spinal versus nonspinal devices (*p* = 0.66), biologic versus nonbiologic devices (*p* = 0.28), and other devices versus non-other devices (*p* = 0.35). The only factor affecting approval was whether the device was an arthroplasty device of any type (*p* = 0.047), though this is no longer significant after multitest correction.

A broad range of topics were queried and debated during panel deliberations, spanning all three categories: outcome measures, randomized trial issues, and issues specific to non-inferiority trials. Within outcome measures, the most frequently debated issue was the assessment of surrogate radiographic endpoints. Questions about the use of plain radiography versus computed tomography, multiple versus single radiologists, and the radiographic assessment of fusion have been active in multiple panel deliberations [6, 7]. Within randomized trial issues, questions of blinding were raised [7, 8], but the most frequently debated issue seemed to be the design of non-inferiority trials. Of the 13 non-inferiority studies in the sample, most had significant debate regarding one or more of the subtopics: choice of active control, the choice of non-inferiority margin, assay sensitivity, and analysis of datasubsets. Anecdotes are offered in the discussion.

## Discussion

The present study demonstrates that the majority of orthopedic devices reviewed by the FDA’s Orthopaedic Devices Panel were studied in pivotal two-arm, non-inferiority trials. The well-known nuances, complexities, and shortcomings in interpreting two-arm non-inferiority trials generated significant debate in multiple panel meetings. This finding is important because the PMA process including panel review is the most intensive scrutiny that a device may receive in consideration of regulatory approval. Similarly, pivotal IDE trials represent the highest level of scientific evidence for a device approved for marketing in the US.

The present study has a number of shortcomings. First, only the subset of IDE trials that were reviewed by the panel were considered since the records of panel meetings are extensive and publicly available. By contrast, the records of PMA applications that do not go to panel are variable. The FDA maintains a database of approved PMA applications [9], but supporting documentation is limited. Most importantly, there is no publicly available database of unapproved PMA applications, which may only be found in SEC filings for publicly listed corporations [10] or by a Freedom of Information Act (FOIA) request for private corporations [11]. Also, identification of clinical trial issues debated at panel is difficult to perform in a way that is simple, clear, and quantitative. Consequently, only a qualitative and anecdotal analysis was possible based on excerpts from a review of the full official transcripts when available.

Non-inferiority studies are designed to demonstrate whether an experimental device is not inferior to an active control device by more than a small margin [12]. This design has a potential ethical advantage since randomizing patients to a sham procedure results in withheld treatment [13]. It also has a potential scientific advantage since powering to show superiority to an effective treatment can require a prohibitive sample size—the size of a non-inferiority trial may be higher than a superiority trial with sham control, but lower than one with active control. Although non-inferiority trials have potential advantages, they are also subject to nuances and complexities of clinical interpretation and data analysis. Some of these are related to similar issues for superiority trials. For example, assay sensitivity is defined as the ability to detect a difference between experiment and control, based on all trial design parameters, outcome measures, and time points. This is a general concept for all trials, but is amplified for non-inferiority trials. A two-arm trial that demonstrates superiority can be interpreted without any additional information, as the assay is proven sensitive by the outcome. By contrast, a two-arm trial that demonstrates non-inferiority has to assume assay sensitivity a priori, since the trial could appear to demonstrate non-inferiority due to an insensitive assay [14]. Approaches to assay sensitivity for non-inferiority trials include a three-arm trial that adds a sham arm [14, 15]; however, all trials during the study period were two-arm. Another approach is to design a trial with similar structure as a previous positive superiority trial; however, the structure includes all endpoints and time points, not just a sensitive clinical outcome measure, which has been debated in panel deliberations [16].

Although multiple issues related to trial design and interpretation recurred during panel deliberations, many were related to two issues unique to non-inferiority trials. The first issue relates to the non-inferiority margin, where the null hypothesis is that the experimental device is inferior to the control by more than the margin [17]. In a typical two-arm trial, the non-inferiority margin is not measured but must be assumed or calculated from prior data, ideally one or more sham-controlled superiority RCTs [15]. For this to occur, the control procedure must be superior to sham and have at least one superiority RCT that allows the effect size of the control procedure to be calculated [14, 18]. Some accepted surgeries have proven themselves over time without this level of scientific support [7, 19], or were tested in an RCT that does not isolate the placebo effect [16]. By contrast, an incorrect non-inferiority margin larger than the effect size of the control could result in an experimental device that is non-inferior to control but not superior to sham—an obvious absurdity and a serious risk. Therefore, the conventional practice of choosing a non-inferiority margin of 10% is at odds with calculating it from prior trials of the active control [16]. A non-inferiority margin of 15% was used in one recent trial, with reference to a precedent trial with a similar margin [20].

The second major issue related to non-inferiority trials was the analysis of datasubsets [17]. Even the best conducted trials have imperfections in randomization, including dropout and crossover [21]. For superiority trials, the ITT principle, in which the data are analyzed as if each patient had received the assigned treatment [14], is regarded as a gold standard because it is statistically conservative, favoring no difference in treatments [22]. However for non-inferiority trials, ITT is not conservative and may be anticonservative, favoring non-inferiority and implying an effect which may not be genuine [23]. The alternatives to ITT—including PP analysis that includes only those patients who complied with randomization and AT analysis that includes patients according to which treatment they received—are controversial in regulatory affairs [24]. To compensate, trials may be analyzed with multiple approaches, including complex analyses such as instrumental variables [25]. For orthopedic devices, the differences between ITT and PP analyses have been discussed in multiple panel deliberations [19, 26].

Regarding device types, a broad range of devices was reviewed at panel including biologic, hip arthroplasty, disc arthroplasty, viscosupplementation, interspinous process devices, and other devices in decreasing order. However, multiple factors affect which device types and devices are reviewed. One is FDA policy regarding the classification of devices as class II or class III, which changed over the study period. For example, in 2003 the FDA convened a panel meeting regarding reclassification of spinal fusion cages from class III devices requiring PMA to class II devices subject to 510(k) premarket notification [27]. Subsequently, no further fusion cages required PMA nor went to panel. The selection bias inherent in studying devices going to panel also extends to individual devices within a class. For example, the first device to come to market went to panel for lumbar arthroplasty [28], cervical arthroplasty [29], and interspinous process devices [30]. But subsequent PMA applications within the same device class were not all reviewed at panel. Regarding the frequency of panel meetings, multiple factors affect this as well, including the rate at which sponsors submit PMA applications and IDE trial data, as well as the rate at which the FDA elects to send PMAs to panel.

Regarding device approval, approximately two-thirds of devices were approved at the time of writing. Arthroplasty devices had the highest rate of approval, and biologic devices had the lowest rate of approval. The only device-related factor associated with approval was whether the device was for arthroplasty; however, this classification represented a diverse group of devices from hip to cervical spine arthroplasty. Of note, it also included a number of hard-on-hard bearing surfaces for hip arthroplasty. Metal-on-metal bearing surfaces have become controversial, and were the subject of a special FDA advisory panel during the study period [31]. Despite this, none of the hip devices that were subsequently recalled or withdrawn from the US market was directly reviewed by the panel [32]. It is true that R3 metal liners (Smith & Nephew, London, UK) were withdrawn during the study period. These were available for use with the Birmingham Hip Resurfacing system and were approved as a supplement to its PMA years after the original Birmingham panel review [33, 34]. But, originally, the R3 system and its predecessors were the subject of a distinct PMA with numerous supplements which did not undergo panel review [35]. Also, the Trident System which was excluded from the present study was recalled, but this was a class II recall due to a change in surgical protocol. [36] In contrast to hip arthroplasty devices, biologic devices had the lowest rate of approval, though this did not reach statistical significance due to the small sample size. Although only two of five biologic devices were initially approved, another device which has undergone years of appeals and re-review may ultimately gained approval [37].

## Conclusion

A broad range of devices is reviewed by the FDA’s Orthopaedic Devices Panel. Although few device or trial-related factors affect approval or recall, panel review is associated with no recent recalls. The majority of devices are studied in pivotal two-arm non-inferiority trials which are subject to complex technical issues and nuances in their interpretation.

## Abbreviations

AT: As-treated
FDA: Food and Drug Administration
FOIA: Freedom of Information Act
IDE: Investigational device exemption
ITT: Intention-to-treat
PMA: Premarket approval
PP: Per-protocol
RCT: Randomized controlled trial
